# Longitudinal Data to Assess Relationships among Plasma Folate, Vitamin B_12_, Non-esterified Fatty Acid, and β-Hydroxybutyrate Concentrations of Holstein Cows during the Transition Period

**DOI:** 10.3390/metabo13040547

**Published:** 2023-04-11

**Authors:** Mélissa Duplessis, Younes Chorfi, Christiane L. Girard

**Affiliations:** 1 Sherbrooke Research and Development Centre, Agriculture & Agri-Food Canada, Sherbrooke, QC J1M 0C8, Canada; 2 Département de biomédecine vétérinaire, Faculté de médecine vétérinaire, Université de Montréal, Saint-Hyacinthe, QC J2S 2M2, Canada

**Keywords:** cobalamin, cattle, folic acid, lipid mobilization

## Abstract

It is well established that the plasma metabolite profile changes during metabolic dysfunction, such as elevated non-esterified fatty acid (NEFA) release when body reserve mobilization is excessive in early lactation cows. Relationships between changes in plasma concentrations of metabolites caused by a metabolic impairment and the status of vitamins, such as folates and vitamin B_12_, have barely been studied in cattle. This study was undertaken to assess relationships between peripartum plasma concentrations of folates, vitamin B_12_, NEFA, and beta-hydroxybutyrate (BHB). Longitudinal data of 48 multiparous Holstein cows from 5 studies were taken from days −14 to 21 relative to calving. Blood samples were taken weekly before calving and either twice or thrice per week postpartum, and plasma was analyzed for folate, vitamin B_12_, NEFA, and BHB concentrations. Postpartum plasma NEFA and BHB concentrations were negatively related to plasma folate concentration at days −14 and −7 relative to parturition, whereas the opposite relationship was noted for the plasma vitamin B_12_:folate ratio. The plasma folate and NEFA areas under the curve from the whole studied period were negatively associated, and the opposite was observed with the association between the plasma vitamin B_12_:folate ratio and NEFA as well as the BHB areas under the curve. The results suggest that there is an increased use of folate for metabolic functions during elevated concentrations of plasma NEFA and BHB. Future research should focus on finding an optimal plasma vitamin B_12_:folate ratio to favor cow health during the challenging period of parturition.

## 1. Introduction

It has been well established that the most challenging period for a dairy cow is the passage from the dry period to lactation, also known as the transition period, from days −21 to 21 relative to calving [1]. At the onset of lactation, to support the increasing energy demand for milk production at the time when dry matter intake (DMI) is not sufficient to fulfil this demand, cows offset this negative energy balance against mobilizing their body reserve. This leads to an augmentation in the plasma non-esterified fatty acid (NEFA) concentration that can be taken up by the liver, which is then either completely oxidized to enter into the Krebs cycle, partially oxidized to ketone bodies such as beta-hydroxybutyrate (BHB), or stored as triacylglycerol [2]. This mechanism is part of a normal adaptation during this period. Nonetheless, it has been shown that detrimental health effects could occur when plasma concentrations of NEFA and BHB are excessive [3].

Plasma folate and vitamin B_12_ concentrations have been reported to vary during the lactation cycle [4] and to be the lowest at parturition and within the first 56 days in milk (DIM), respectively [5,6]. These two vitamins are closely related in the metabolism. Indeed, 5-methyl-tetrahydrofolate, a form of folates, can transfer its methyl group to the coenzyme vitamin B_12_ to convert homocysteine to methionine [7]. Methionine can be used in protein synthesis or be activated as S-adenosylmethionine, which is the methyl donor for more than 100 methyltransferases, including the one involved in DNA methylation [7]. Vitamin B_12_ also acts as a coenzyme for methylmalonyl CoA mutase in the conversion of methylmalonyl CoA to succinyl CoA, to allow its entry into the Krebs cycle, a key step of propionate degradation, which is the major source of glucose for ruminants [8]. Due to their important roles and the decrease of their plasma concentrations around calving, previous studies have evaluated the effect of folate and vitamin B_12_ supplementation during the transition period on energy metabolism and lactation performance [9,10,11]. These studies concluded that the supplementation, increasing folate and vitamin B_12_ plasma concentrations, altered energy partitioning during the transition period, either by lowering plasma NEFA with similar milk yield and DMI or by increasing milk production with similar DMI and similar or lower plasma NEFA. Duplessis, et al. [12] reported an increase of milk yield by 13.5% with folic acid and vitamin B_12_ supplementation coupled with a slight augmentation of plasma NEFA concentration, with no change in plasma BHB concentration. These two vitamins are synthesized by rumen bacteria, and their apparent ruminal synthesis is affected by dietary management [13]; therefore, the plasma folate and vitamin B_12_ concentrations of cows not receiving folic acid and vitamin B_12_ supplementation vary among the population [4]. It is not known if the natural variation in folate and vitamin B_12_ status can have an impact on plasma metabolites related to energy metabolism, such as plasma NEFA and BHB concentrations, or vice versa.

In their review, Overton, et al. [14] stressed the importance of studying plasma metabolite changes to monitor the metabolic function and health of dairy cows. Most research has focused on how blood NEFA and BHB can be used as metabolic health indicators during the transition period [14]. Other metabolites, such as vitamins, have been less studied [15]. Recently, in a cross-sectional study, it was suggested that plasma vitamin B_12_ and NEFA concentrations were positively associated in early lactating cows (Spearman rank correlation coefficients from 0.36 to 0.53) [16]. Our hypothesis was that plasma folate, vitamin B_12_, NEFA, and BHB concentrations are associated during the transition period. This study was undertaken to assess relationships between peripartum plasma concentrations of folates, vitamin B_12_, NEFA, and BHB using longitudinal data from five different studies. The impact of prepartum plasma folate and vitamin B_12_ concentrations on postpartum plasma NEFA and BHB concentrations was also evaluated.

## 2. Materials and Methods

### 2.1. Dataset

Data from cows not receiving vitamin supplementation from 5 studies conducted either at the dairy herd of Agriculture and Agri-Food Canada Research Centre [Sherbrooke, QC, Canada [9,12,17]] or at the dairy herd of the Center for Research in Animal Science [Deschambault, QC, Canada [18,19]] were used in the present study. These studies were first designed to evaluate the impact of B-vitamin supplementation during the transition period and early lactation on energy metabolism. Before the onset of the experiments, all protocols were revised by the Animal Care Committees of either the Sherbrooke Research and Development Centre or Université Laval to comply with the guidelines provided by the Canadian Council on Animal Care [20]. The dataset included a total of 48 multiparous Holstein cows [Study 1, *n* = 12 [19]; Study 2, *n* = 12 [18]; Study 3, *n* = 10 [17]; Study 4, *n* = 6 [9]; Study 5, *n* = 8 [12]] housed in tie stall and milked twice daily. The number of cows involved in each study varied according to the specific objectives and treatments used in these studies. Cows weighed on average 726 (SE: 0.3) kg 14 days prior to parturition. Before calving, cows were fed a precalving total mixed ration (TMR) twice daily for studies conducted at Sherbrooke and once daily for studies conducted at Deschambault, and after calving, a lactation TMR once daily for all experiments.

### 2.2. Data Collection and Analyses

The quantity of TMR served and orts were recorded daily. Milk yield was recorded at each milking. Blood samples were obtained from coccygeal venipuncture (Vacutainer system, Becton, Dickinson, and Co., Franklin Lakes, NJ, USA) with EDTA tubes every week before parturition and either twice in studies 1, 2, and 4 or thrice in studies 3 and 5 per week within the first 3 weeks after calving. Plasma folate, vitamin B_12_ (SimulTRAC B_12_/FOLATE-S, MP Biomedicals, Solon, OH, USA), NEFA [HR Series NEFA-HR(2), Wako Chemicals USA Inc., Richmond, VA, USA], and BHB (β-hydroxybutyrate reagent set, Pointe Scientific Inc., Canton, MI, USA) concentrations were analyzed with commercial kits, as described by Duplessis et al. [9]. Plasma folate and vitamin B_12_ concentrations were analyzed at each blood collection timepoint in studies 1, 2, and 4 and weekly in studies 3 and 5. All plasma samples collected were analyzed for NEFA and BHB concentrations in all studies.

### 2.3. Calculations and Statistical Analyses

A dataset standardization needed to be conducted before analyses as there were slight methodology differences among the 5 studies. Only data from days −14 to 21 relative to calving were considered in the statistical analyses as one study did not have data prior to 14 days before parturition. Except for area under the curve (AUC) calculation, only 2 postpartum data per week for plasma NEFA and BHB concentrations were kept for further analysis, to homogenize the dataset. Indeed, as plasma samples were taken thrice weekly after calving in studies 3 and 5, a total of 3 postpartum samples per cow were dropped (i.e., the one closest to calving, 7, and 14 DIM). Only weekly samples were taken for presenting descriptive statistics. Vitamin B_12_ to folate ratio was calculated because of the close interrelationship of these 2 vitamins in the methylation cycle [7]. Daily data of DMI and milk yield were weekly averaged. Minimum and maximum plasma folate and vitamin B_12_ concentrations as well as the DIM at which these concentrations were reached were obtained with Proc SQL of SAS [version 9.4, SAS Institute, Cary, NC, USA [21]]. Plasma folate and vitamin B_12_ concentration variations during the whole studied period were calculated as: the highest–the lowest vitamin concentrations. Taking all available records from −14 to 21 DIM, total AUC was calculated according to Cardoso, et al. [22] for plasma folate, vitamin B_12_, vitamin B_12_:folate ratio, NEFA, and BHB concentrations. Six cows were deleted from the AUC analysis as plasma analyses from day 14 precalving were missing. Prepartum and postpartum elevated plasma NEFA thresholds were, respectively, 0.3 and 0.7 m*M*, and the thresholds of precalving and postcalving hyperketonemia were, respectively, set at 0.6 and 1.0 m*M* of BHB according to Ospina, et al. [3].

Proc MIXED of SAS with repeated measurements in time was used to obtain weekly averages of DMI, BW, plasma metabolite concentrations, separately for pre- and postpartum periods, and milk yield. Time was the fixed effect for these models. Similar multilevel models with repeated measurements in time were used to study the relationship between postpartum plasma NEFA and BHB concentrations and prepartum plasma folate and vitamin B_12_ concentrations or variation throughout the studied period. Time and vitamin concentration variations, vitamin statuses at days −14 or −7 before calving as continuous variables were fixed effects. In all cases, cows nested within studies was considered the random effect. Several covariance structures were tested: compound symmetry, heterogeneous compound symmetry, first-order autoregressive, heterogeneous first-order autoregressive, Toeplitz, heterogeneous Toeplitz, first-order ante dependence, and unstructured, and the one leading to the smallest Bayesian Information Criterion was chosen. The normality and homoscedasticity of residuals were graphically assessed. Proc UNIVARIATE of SAS was used to evaluate if the AUC distribution of the studied variables was normal using the Kolmogorov–Smirnov Goodness-of-Fit test. The vitamin B_12_:folate ratio, NEFA, and BHB AUCs were not normally distributed. Therefore, Proc CORR of SAS was used to obtain Spearman correlation coefficients between AUCs from different plasma metabolites. Significance was considered when *p*-values ≤ 0.05 and at a tendency of *p* ≤ 0.10.

## 3. Results

### 3.1. Descriptive Statistics

Weekly milk yield, DMI, and plasma metabolite concentrations from days −14 to 21 relative to parturition are shown in Table 1. For most variables, there was a significant variation or a tendency in relation to the calving date (Time effect, *p* ≤ 0.09). Table 2 presents the distribution of the lowest and the highest plasma folate and vitamin B_12_ concentrations and their ratio as well as the day of the event relative to calving and their variation over the whole studied period. It is worth noting that the lowest and the highest plasma folate concentrations were, respectively, observed on average at d 0.8 and 10.9 after calving, and the opposite was noted regarding plasma vitamin B_12_ concentration and plasma vitamin B_12_:folate ratio. There were 23 prepartum plasma samples out of 86 from 17 cows and 35 postpartum plasma samples out of 232 from 18 cows with elevated NEFA. Prepartum plasma samples suggesting hyperketonemia included 46 samples out of 86 from 28 cows, whereas it was 89 samples out of 232 from 31 cows for the postpartum period.

### 3.2. Relationships among Plasma Folates, Vitamin B_12_, NEFA, and BHB

There was no relationship between plasma folate concentration variations from −14 to 21 DIM and postpartum plasma NEFA and BHB concentrations (*p* ≥ 0.11; Table 3). Postpartum plasma NEFA and BHB concentrations were negatively related with plasma folate concentration at days −14 and −7 relative to parturition (*p* < 0.0001). No significant association was obtained for plasma vitamin B_12_ concentration (Table 4). Postpartum plasma NEFA and BHB concentrations were negatively related to plasma vitamin B_12_:folate ratio variation from days −14 to 21 DIM (*p* ≤ 0.01; Table 5). Postpartum plasma NEFA and BHB concentrations were positively associated with plasma vitamin B_12_:folate ratios at days −14 and −7 relative to calving (*p* ≤ 0.06).

As expected, the plasma folate and vitamin B_12_ AUCs had a significant relationship with their ratio AUC (*p* ≤ 0.0002; Table 6). Interestingly, the plasma folate and NEFA or BHB AUCs were negatively associated, and the opposite was observed for the association between the plasma vitamin B_12_:folate ratio and NEFA and BHB AUCs (*p* ≤ 0.003). A Spearman correlation coefficient of 0.38 was obtained for the NEFA and BHB AUC relationship (*p* = 0.01; Table 6).

**Table 6:** Spearman rank correlation coefficients (*p*-values in parentheses) between peripartum plasma folate, vitamin B_12_, NEFA, and BHB concentration AUCs ^1^.

Item	Plasma Folate AUC	Plasma Vitamin B_12_ AUC	Vitamin B_12_:Folate Ratio AUC	NEFA AUC	BHB AUC
Plasma folate AUC	1.00	0.17 (0.29)	−0.66 (<0.0001)	−0.60 (<0.0001)	−0.35 (0.02)
Plasma vitamin B_12_ AUC		1.00	0.54 (0.0002)	0.10 (0.52)	0.15 (0.35)
Vitamin B_12_:folate ratio AUC			1.00	0.66 (<0.0001)	0.45 (0.003)
NEFA AUC				1.00	0.38 (0.01)
BHB AUC					1.00

^1^ AUC calculated from days −14 to 21 relative to calving data according to Cardoso, et al. [22]. Abbreviations: AUC = area under the curve; BHB = beta-hydroxybutyrate; NEFA = non-esterified fatty acid.

## 4. Discussion

Averaged pre- and postpartum plasma BHB concentrations were greater than the threshold value known to cause adverse health effects to early lactation dairy cows [0.6 mmol/L before calving and 1.0 mmol/L after calving; 3]; whereas averaged pre- and postpartum plasma NEFA concentrations were lower than the lowest threshold value [0.3 mmol/L before calving and 0.7 mmol/L after calving; 3]. Plasma folate and vitamin B_12_ concentration patterns around calving in the current study were similar to those previously reported [6,23,24]. Longitudinal data with frequent samplings, as used in the current assessment, allowed us for the first time to describe the day of the lowest and highest plasma folate and vitamin B_12_ concentrations and their ratio, along with their respective concentrations. Results suggested that the lowest and the highest plasma folate and vitamin B_12_ concentrations can be observed at any time from −14 to 21 DIM. Interestingly, the average DIM at which the lowest and the highest plasma folate concentrations were reached were those when, at the opposite, the highest and the lowest plasma concentrations of vitamin B_12_ were observed. Nevertheless, Duplessis, et al. [4] demonstrated that it was possible to maximize plasma concentrations of both vitamins and that the correlation between the two vitamins was weak. Accordingly, the relationship between the plasma folate and vitamin B_12_ AUCs was not significant around calving in the current study. To our knowledge, this is the first time that the plasma vitamin B_12_:folate ratio has been reported in dairy cows. As previously mentioned, these two vitamins are interrelated in the metabolism. Known as the methyl folate trap, a lack of vitamin B_12_ can cause the accumulation of 5-methyl-tetrahydrofolate, hence reducing other active forms of folates [7]. Thus, the objective behind including the vitamin B_12_:folate ratio in the analyses was to evaluate if the ratio, rather than the two vitamins taken separately, best described the relationship with plasma NEFA and BHB concentrations. In humans, Muro, et al. [25] concluded that the ratio between these two vitamins could potentially be used in the differential diagnosis of liver disease.

Prepartum plasma folate concentrations were negatively related with postpartum plasma NEFA and BHB concentrations. It is noteworthy that although there was no relationship with prepartum vitamin B_12_ concentration, the prepartum vitamin B_12_:folate ratio was positively associated with postpartum plasma NEFA and BHB concentrations. Moreover, the ratio variation during the transition period was negatively associated with postpartum plasma NEFA and BHB concentrations. This indicates that an optimal plasma ratio of these two vitamins before calving is one important factor for ensuring a cow’s health after calving. More research is needed to evaluate which ratio should be targeted and under which conditions it can be reached. To our knowledge, these associations were reported for the first time, and further investigations should be carried out to confirm their biological and clinical relevance.

It has been reported in humans that plasma folate and vitamin B_12_ concentrations are, respectively, lower and greater in those suffering from liver disease [25,26]. In dairy cows, Obitz and Fürll [27] concluded that high serum vitamin B_12_ in early lactation could indicate a health issue. Duplessis, et al. [16] also obtained a positive correlation coefficient between postpartum plasma vitamin B_12_ and NEFA concentrations in dairy cows. In opposition, in the study of Corse and Elliot [28], 3 cows out of 12 had hyperketonemia at 3 weeks postpartum, and those cows had the lowest prepartum serum vitamin B_12_ concentrations. Surprisingly, in the present assessment, the plasma vitamin B_12_ AUC was not related with the NEFA or BHB AUCs. This could be due to the different studied period length, i.e., longitudinal data from days −14 to 21 relative to calving vs. one postpartum timepoint. Consistent with the results in humans cited above regarding plasma folates and liver disease, the plasma folate and NEFA or BHB AUCs were negatively related. In the current study, it was not possible to evaluate liver disease, but it is well known that elevated plasma NEFA and BHB concentrations can be associated to liver diseases such as fatty liver [29]. Młodzik-Czyżewska, et al. [30] also found a negative association between serum folate and some biomarkers of lipid metabolism in humans. One possible explanation might be an increased use of folates with increased circulating NEFA due to the role of folates in methyl group transfer, which is needed to synthesize the very-low-density lipoprotein used to export the liver triacylglycerol formed during hepatic NEFA accumulation [31]. Increasing methyl group supply also promotes carnitine synthesis to support the β-oxidation of long-chain fatty acids released during body reserve mobilization [32]. Consequently, Girard and Duplessis [33] hypothesized that folates might have a role in the maintenance of an efficient utilization of long-chain fatty acids such as NEFA through an enhancement of their β-oxidation. Interestingly, correlation coefficients between vitamin B_12_:folate ratio and NEFA or BHB AUCs were higher than for the plasma folate AUC alone, suggesting again that an optimal ratio of these two vitamins is necessary. The correlation coefficient obtained between the plasma NEFA and BHB concentration AUCs was slightly greater in the current study than that previously reported during the same timeframe [34].

## 5. Conclusions

Longitudinal data from five previous studies were used to gather novel knowledge on folates, vitamin B_12_, NEFA, and BHB metabolism during the transition period in dairy cows. Links with prepartum plasma folate and vitamin B_12_:folate ratio statuses, and vitamin B_12_:folate variation with postpartum plasma NEFA and BHB concentrations, have been reported for the first time. Negative relationships between plasma folate and NEFA and BHB AUCs could indicate a greater use of folates for metabolic functions as plasma NEFA and BHB concentrations rise. The results on the plasma vitamin B_12_:folate ratio suggest that an optimal ratio of these two vitamins should be targeted to optimize cow health, but further studies need to be conducted in this regard.

## Figures and Tables

**Table 1:** Weekly averages of DMI, plasma metabolite concentrations, and milk yield from days −14 to 21 relative to calving of 48 multiparous Holstein dairy cows.

Variable	Days Relative to Calving			SEM	*p*-Value
Precalving	−14	−7			
DMI (kg/d)	13.3	12.6	-	0.3	0.002
Plasma folates (ng/mL)	11.1	11.6	-	0.7	0.21
Plasma vitamin B_12_ (pg/mL)	238.6	268.6	-	17.6	0.09
Plasma vitamin B_12_:folate ratio	23.9	26.5		1.9	0.18
Plasma NEFA (m*M*)	0.20	0.27	-	0.02	0.003
Plasma BHB (m*M*)	0.68	0.66	-	0.03	0.61
Variable	Days relative to calving			SEM	*p*-value
Postcalving	7	14	21		
Milk yield (kg/d)	30.5	37.2	40.8	1.2	<0.0001
DMI (kg/d)	16.3	19.7	21.3	0.6	<0.0001
Plasma folates (ng/mL)	12.3	12.3	13.7	0.8	0.004
Plasma vitamin B_12_ (pg/mL)	223.0	197.9	191.7	15.1	0.09
Plasma vitamin B_12_:folate ratio	20.3	19.0	16.9	1.8	0.05
Plasma NEFA (m*M*)	0.44	0.35	0.31	0.03	<0.0001
Plasma BHB (m*M*)	1.07	1.36	1.14	0.15	0.07

Abbreviations: BHB = beta-hydroxybutyrate; DMI = dry matter intake; NEFA = non-esterified fatty acid.

**Table 2:** Descriptive statistics of the lowest and the highest days of the event, and variation of plasma folate and vitamin B_12_ concentrations observed in 48 multiparous Holstein dairy cows from days −14 to 21 relative to calving.

Variable	Mean	SD	Minimum	Maximum
Plasma folates				
Lowest concentration				
Day relative to calving	0.8	11.0	−14	21
Concentration (ng/mL)	9.73	3.96	4.75	19.13
Highest concentration				
Day relative to calving	10.9	9.5	−12	21
Concentration (ng/mL)	15.71	4.99	7.65	30.91
Variation ^1^ (ng/mL)	5.98	2.69	1.13	12.05
Plasma vitamin B_12_				
Lowest concentration				
Day relative to calving	10.5	9.4	−13	21
Concentration (pg/mL)	155.9	51.4	27.4	288.4
Highest concentration				
Day relative to calving	0.4	8.7	−14	21
Concentration (pg/mL)	313.0	131.2	153.3	791.2
Variation ^1^ (pg/mL)	157.1	119.7	42.4	627.7
Plasma vitamin B_12_:folate ratio				
Lowest ratio				
Day relative to calving	11.1	8.8	−13	21
Ratio	12.3	5.3	3.9	27.7
Highest ratio				
Day relative to calving	−0.6	9.2	−14	20
Ratio	32.0	14.6	14.7	64.1
Variation ^1^	19.7	12.5	4.9	48.2

^1^ Computed as the highest–the lowest vitamin concentrations obtained from days −14 to 21 relative to calving.

**Table 3:** Relationships between postpartum plasma NEFA and BHB concentrations ^1^ and prepartum plasma folate concentration and its variation from −14 to 21 DIM.

Variable	Plasma NEFA			Plasma BHB		
	Estimate	SE	*p*-Value	Estimate	SE	*p*-Value
Plasma folate variation ^2^						
Intercept	0.42	0.07	<0.0001	0.64	0.08	<0.0001
Plasma folate concentration	0.02	0.01	0.11	0.002	0.012	0.90
DIM			<0.0001			0.28
Plasma folates at days −14 before calving						
Intercept	0.83	0.07	<0.0001	1.54	0.22	<0.0001
Plasma folate concentration	−0.03	0.006	<0.0001	−0.05	0.02	0.01
DIM			<0.0001			0.41
Plasma folates at days −7 before calving						
Intercept	0.72	0.08	<0.0001	1.55	0.21	<0.0001
Plasma folate concentration	−0.02	0.006	0.004	−0.05	0.02	0.003
DIM			<0.0001			0.41

^1^ Postpartum data from 0 to 21 DIM included. ^2^ Computed as the highest–the lowest vitamin concentrations obtained from days −14 to 21 relative to calving. Abbreviations: BHB = beta-hydroxybutyrate; DIM = days in milk; NEFA = non-esterified fatty acid.

**Table 4:** Relationships between postpartum plasma NEFA and BHB concentrations ^1^ and prepartum plasma vitamin B_12_ concentration and its variation from −14 to 21 DIM.

Variable	Plasma NEFA			Plasma BHB		
	Estimate	SE	*p*-Value	Estimate	SE	*p*-Value
Plasma vitamin B_12_ variation ^2^						
Intercept	0.52	0.05	<0.0001	1.04	0.13	<0.0001
Plasma vitamin B_12_ concentration	−0.00004	0.0002	0.85	−0.0004	0.0006	0.58
DIM			<0.0001			0.28
Plasma vitamin B_12_ at days −14 before calving						
Intercept	0.57	0.08	<0.0001	1.13	0.21	<0.0001
Plasma vitamin B_12_ concentration	−0.0001	0.0003	0.69	−0.0004	0.0008	0.58
DIM			<0.0001			0.42
Plasma vitamin B_12_ at days −7 before calving						
Intercept	0.48	0.08	<0.0001	0.88	0.20	<0.0001
Plasma vitamin B_12_ concentration	0.0002	0.0002	0.52	0.0005	0.0007	0.49
DIM			<0.0001			0.43

^1^ Postpartum data from 0 to 21 DIM included. ^2^ Computed as the highest–the lowest vitamin concentrations obtained from days −14 to 21 relative to calving. Abbreviations: BHB = beta-hydroxybutyrate; DIM = days in milk; NEFA = non-esterified fatty acid.

**Table 5:** Relationships between postpartum plasma NEFA and BHB concentrations ^1^ and prepartum plasma vitamin B_12_:folate ratio and its variation from −14 to 21 DIM.

Variable	Plasma NEFA			Plasma BHB		
	Estimate	SE	*p*-Value	Estimate	SE	*p*-Value
Plasma vitamin B_12_:folate ratio variation ^2^						
Intercept	0.72	0.08	<0.0001	1.54	0.22	<0.0001
Plasma vitamin B_12_:folate ratio	−0.02	0.006	0.004	−0.05	0.02	0.01
DIM			<0.0001			0.41
Plasma vitamin B_12_:folate ratio at days −14 before calving						
Intercept	0.35	0.07	<0.0001	0.71	0.19	0.0008
Plasma vitamin B_12_:folate ratio	0.008	0.002	0.0008	0.01	0.007	0.06
DIM			<0.0001			0.41
Plasma vitamin B_12_:folate ratio at days −7 before calving						
Intercept	0.35	0.06	<0.0001	0.38	0.21	0.08
Plasma vitamin B_12_:folate ratio	0.006	0.002	0.0004	0.02	0.007	0.0009
DIM			<0.0001			0.12

^1^ Postpartum data from 0 to 21 DIM included. ^2^ Computed as the highest–the lowest ratio obtained from days −14 to 21 relative to calving. Abbreviations: BHB = beta-hydroxybutyrate; DIM = days in milk; NEFA = non-esterified fatty acid.

## Data Availability

The data presented in this study are available on request from the corresponding author. The data are not publicly available due to privacy.
